# Exophthalmic Goitre

**Published:** 1907-03

**Authors:** 


### EXOPHTHALMIC GOITRE.

James Ewing, in the New York Medical Journal for December 8, 1906, points out the limitations of serum therapy in Graves's disease. While there is a good theoretical basis for the use of antitoxic serum therapy in Graves's disease. While there is a good theoretical basis against the thyroglobin, and a somewhat less certain outlook for the action of an antibody prepared against the nucleo-proteid of the thyroid gland, there are many considerations pointing to extensive limitations in the use of such a serum in all stages of the disease. He states that it is by no means certain that the thyroid gland is the sole primary source of disturbance in the disease, and that whatever may prove to be the connection between constitutio lymphatica with its enlarged thymus, and Graves's disease, the existence of this condition in the majority of fatal cases stands as an obscure factor, which may escape the influence of a serum directed against the thyroid. The lesions in the thyroid itself in the advanced cases of the disease seem to constitute a more serius limitation to serum therapy. With the extensive changes and degenerations present in such cases, it is difficult to see how any therapeutic agent can permanently affect the disordered function of the gland. A serum treatment designed to influence the earlier stages of Graves's disease, if really effective in those stages, must become less and less potent as the character of the morbid process changes, and in the latter stages of prolonged cases it may even do harm. The serum treatment must apparently be adjusted to each case and stage of the disease, and this requirement opens up a new and practical field for the clinical and chemical study of the disease. Finally, in Graves's disease, which is so often associated with the other functional and organic diseases, the action of a serum can be safely judged only in earlier or uncomplicated cases. In the later stages so many self perpetuating functional and organic sequelæ arise that effective influence over the original disorder seems much less likely to be secured.

